# In Vitro Selection of Antibodies Targeting *Yersinia pestis* Membrane Lipids Using Nanodisc-Based Antigen Presentation

**DOI:** 10.3390/pathogens15060651

**Published:** 2026-06-20

**Authors:** Madeline R. Bolding, Sarah C. Mozden, Olivia R. Pimentel, Makaela M. Montoya, Jessica Z. Kubicek-Sutherland, Nileena Velappan

**Affiliations:** 1 Bioscience Division, Los Alamos National Laboratory, Los Alamos, NM 87545, USA; 2 Chemistry Division, Los Alamos National Laboratory, Los Alamos, NM 87545, USAjzk@lanl.gov (J.Z.K.-S.)

**Keywords:** lipid antigens, antibody selection, nanodiscs, phage display, yeast display, *Yersinia pestis*, lipopolysaccharide, scFv antibodies

## Abstract

Proteins are the most common targets for antibody discovery and vaccine development, but their sequence variability can limit the breadth of resulting antigens. Lipids represent an alternative class of antigens due to their structural conservation and roles in host–pathogen interactions. Here, we describe the development and optimization of an in vitro antibody selection workflow using lipid-containing nanodiscs as antigen presentation platforms to enable phage and yeast display selections under conditions adapted for these non-protein targets. Lipopolysaccharide (LPS) nanodiscs were first used as a model system to evaluate selection strategies, including competitive and subtractive approaches to reduce non-specific binders, yielding peptide and single-chain variable fragment (scFv) binders that were affinity matured to improve binding signals. The same approach was subsequently used to select scFv antibodies that recognize lipid nanodiscs prepared from *Yersinia pestis* membrane lipid extracts. These antibodies show binding to lipid nanodiscs derived from *Y. pestis*, with evidence of selectivity relative to control nanodiscs. Overall, this work establishes a workflow for antibody selection against lipid-containing nanodisc antigens and highlights practical considerations associated with these targets. The approach may be useful for generating affinity reagents to membrane-associated lipids, although further characterization is required to define antigen specificity and functional activity.

## 1. Introduction

*Yersinia pestis* is a Gram-negative bacterium that causes plague, a severe and potentially fatal vector-borne zoonotic disease [1]. The bacterium is persistent in contaminated soil and infects burrowing animals before being transmitted by flea bites to humans, who serve as incidental hosts [1,2]. *Yersinia* species are widely distributed across environmental and animal reservoirs, contributing to transmission complexity and strain diversity [3]. Though only one bacterial agent causes plague, the disease can manifest in three clinical forms: bubonic plague, pneumonic plague, and septicemic plague. All forms are fatal in the absence of timely antibiotic treatment [4]. Multiple plague pandemics have been reported in recorded history, and the use of evolved or engineered *Y. pestis* as a bioweapon remains a significant biosecurity concern [5].

Diagnostic tests for plague include culturing from bubo aspirates, blood, or sputum followed by microscopy analysis using Gram, Wright, Giemsa, or Wayson stains of smears showing characteristic bipolar-staining “safety pin” rods [6]. Highly sensitive polymerase chain reaction (PCR) assays targeting *pla*, *caf1* (Fraction 1, F1 antigen), or *yopM* genes have also been used for rapid diagnosis [7]. Immunological tests targeting F1 and low-calcium response V antigen (LcrV), the dominant surface antigens of *Y. pestis,* using enzyme- or fluorescence-linked immunoassays are routinely used for dipstick-based diagnostic assays [8].

The successful treatment of plague depends on prompt recognition and the timely use of effective antibiotics. Most reported strains of *Y. pestis* are susceptible to streptomycin. However, the emergence (natural or engineered) of multidrug-resistant strains is of concern and motivates the development of novel diagnostics, vaccines, and treatment options [9]. Similar to current diagnostic approaches, existing vaccine strategies focus on the immunodominant proteins F1 and LcrV, delivered either as recombinant subunits or expressed in *Yersinia pseudotuberculosis*, the progenitor of *Y. pestis*, to generate a live attenuated plague vaccine [5]. Subunit and nanoparticle-based formulations incorporating these antigens have also been explored as alternative vaccine strategies [10]. However, the F1 capsular antigen is not expressed under all conditions and can be absent in certain strains, which may limit the effectiveness of F1-targeted approaches [11]. Alternate treatment strategies include passive antibody therapies targeting these immunodominant proteins [12]. These antibodies can target different epitopes on F1 and/or LcrV proteins. The effectiveness of these antibodies may be improved by drug conjugates such as toxic moieties or radioactive compounds as reported in previous studies [8,13,14]. However, protein sequences are often strain-specific and subject to sequence variation over time [15]. This variability poses a significant challenge for the durability and breadth of protein-targeted countermeasures and underscores the need to consider alternative antigen classes.

Lipids represent an alternative class of antigens due to their relative structural conservation and roles in host–pathogen interactions [16,17,18]. However, lipids have been associated with limited adaptive antibody responses despite their highly immunostimulatory effects on the innate immune system [19,20]. This discrepancy may be explained by studies showing that lipid presentation in the context of carrier molecules can alter immune recognition [21,22]. Bacterial glycolipids including lipopolysaccharide (LPS) from Gram-negatives, lipoteichoic acid from Gram-positives, and lipoarabinomannan from mycobacteria are recognized by host receptors and are known to elicit antibody responses under certain presentation contexts [23,24,25]. These observations motivate the development of antigen presentation strategies that enable antibody selection against lipid-containing targets.

Nanodiscs are lipoprotein particles that mimic human high-density lipoproteins (HDLs) and contain a discoidal lipid bilayer stabilized by a membrane scaffold protein [26]. The membrane scaffold protein is a derivative of the human apolipoprotein AI and can be used to facilitate immobilization of nanodiscs for antibody selection workflows. The lipid bilayer component of the nanodisc holds the bacterial lipids in a membrane-like configuration that may facilitate antibody recognition. This format provides a potential approach for presenting lipid antigens in vitro.

Phage display has been established as an effective method for in vitro antibody selection [27]. In combination with yeast display, this technology has been successful in selecting specific antibodies to various targets of interest [28]. These display technologies allow carefully controlled selection and screening conditions using various antigen presentation formats. These in vitro methods also overcome immunological tolerance and allow selection of affinity reagents to highly conserved epitopes. Using these techniques, we have selected and characterized antibodies to mammalian antigens, bacterial and viral cell membrane proteins [8,29,30], and small molecule targets such as post-translational modifications [31,32]. Thus, we sought to apply these approaches to the selection of antibodies targeting lipid-containing nanodisc antigens.

Here we describe the antigen design strategies for phage and yeast display-based in vitro antibody selection using lipid nanodiscs. We initially optimized the selection methodology using nanodiscs containing *Escherichia coli* LPS as a model antigen. Selected binders were further subjected to affinity maturation using error-prone PCR and flow cytometry-based sorting. The optimized protocol was subsequently used to select antibodies to nanodiscs prepared from *Y. pestis* cell membranes. We report the identification and initial characterization of peptide and single-chain variable fragment (scFv) binders to LPS-containing nanodiscs, as well as scFv antibodies that recognize nanodiscs derived from *Y. pestis* membrane lipid extracts. These results establish a workflow for antibody selection against lipid-containing nanodisc antigens and provide a foundation for further development and characterization of lipid-targeting binders.

## 2. Materials and Methods

### 2.1. Bacterial Growth, Lipid Extraction, and Nanodisc Formulation

Bacterial growth, total lipid extraction, and nanodisc formulation were performed as previously described [33], with minor modifications.

Bacterial growth and lipid extraction: *Yersinia pestis* strain A1122 (BEI Resources, NR-636) is excluded from Select Agent regulations and was handled under biosafety level 2 (BSL-2) conditions in accordance with institutional guidelines. Bacterial cultures were initiated from single colonies grown on tryptic soy agar plates (TSA; VWR 100217–300) at 28 °C for 24–48 h and inoculated into liquid tryptic soy broth (TSB; BD Bacto 211825, Fisher scientific, Waltham, MA, USA) in 100–150 mL volumes in 500 mL flasks. Cultures were grown at 28 °C with shaking at 150 rpm for 24–48 h to stationary phase, as determined by optical density at 600 nm. Cells were harvested by centrifugation and total lipids were extracted from bacterial pellets using a modified Bligh and Dyer method [34], and lipid extracts were stored at −20 °C following confirmation of non-viability.

Nanodisc assembly: Nanodiscs were assembled using membrane scaffold protein MSP1D1 and total lipid extracts as described previously with modifications [33]. Control nanodiscs (Cnd) were composed of 1,2-dimyristoyl-sn-glycero-3-phosphocholine (DMPC; Sigma P2663, St. Louis, MO, USA), while *Y. pestis* nanodiscs were prepared using total lipid extracts. Lipopolysaccharide (LPS) nanodiscs were assembled from a mixture of *Escherichia coli* O11:B4 LPS (Sigma, L2630) and DMPC (10:90 *v*/*v*). Lipids were dissolved in chloroform, dried overnight, and resuspended in cholate buffer (26 mM sodium cholate; Sigma C6445) to a final concentration of 10 mg/mL. Samples were sonicated for 15 min at 25 °C, vortexed, and incubated on an orbital shaker for 30 min at room temperature to ensure complete solubilization. MSP1D1 (Sigma MSP01, 5 mg/mL) or biotinylated MSP1D1 (Sigma MSP13, 5 mg/mL) was added to the lipid extracts in a glass tube and incubated at 25 °C for 1 h.

Nanodisc purification: Nanodiscs were purified by detergent removal via dialysis using 6–8 kDa molecular weight cutoff cassettes (Sigma PURD60100) in detergent-free buffer (100 mM NaCl, Tris-HCl, pH 8) with three buffer exchanges over 24 h. Following dialysis, control nanodiscs were clarified by centrifugation (45 min at 13,000× *g*), while LPS- and *Y. pestis*-derived nanodiscs were further purified by size exclusion chromatography.

Fluorescent labeling: Fluorescent nanodiscs were generated by labeling purified assemblies with Alexa Fluor 633 NHS ester (Invitrogen A20105) according to the manufacturer’s protocol.

Nanodisc characterization: Nanodiscs were characterized by dynamic light scattering (DLS), native gel electrophoresis, and transmission electron microscopy to assess size and structural integrity. Only nanodiscs with diameters in the range of 9–12 nm were used for downstream experiments. Nanodrop spectroscopy was used to estimate concentration and confirm fluorophore incorporation. Lipid incorporation and composition were verified by liquid chromatography–mass spectrometry. Detailed lipidomic characterization of the *Y. pestis* nanodisc preparations, including analysis of lipid composition and heterogeneity, has been performed and will be reported separately. Detailed protocols for nanodisc characterization have been previously described [33].

### 2.2. ScFv Antibody Selections by Phage Display

**Figure 1:**
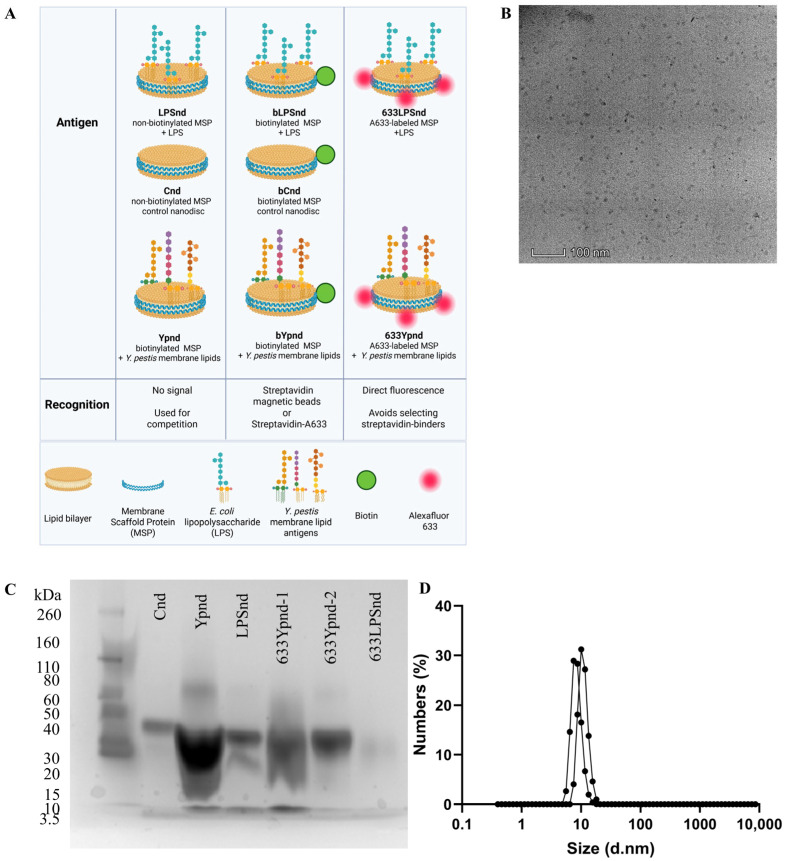
Lipid nanodisc antigen preparation. (**A**) Schematic representation of nanodisc antigen formats and their use in in vitro antibody selection, created in BioRender (Velappan, N., 2026; https://BioRender.com/fxrydrh). Control nanodiscs (Cnd), lipopolysaccharide-containing nanodiscs (LPSnd), and *Yersinia pestis* membrane lipid nanodiscs (Ypnd) were prepared with non-biotinylated, biotinylated, or Alexa Fluor 633 (A633)-labeled membrane scaffold protein (MSP) to enable competition, bead-based capture, or direct fluorescence detection, respectively. (**B**) Negative stain transmission electron micrographs of nanodiscs prepared from *Y. pestis* membrane lipids (Ypnd). Samples were applied to glow-discharged copper grids and negatively stained with UranyLess (Electron Microscopy Sciences, 22409). Images were acquired using a Talos L120C transmission electron microscope at 120 kV. Scale bar, 100 nm. (**C**) Native polyacrylamide gel electrophoresis analysis of nanodisc formulations. For 633Ypnd-1, MSP was labeled with A633 prior to nanodisc assembly. For 633Ypnd-2, fluorophore conjugation was performed after nanodisc assembly. (**D**) Dynamic light scattering (DLS) analysis of *Y. pestis* nanodiscs (Ypnd), showing particle size distribution by number (%).

**Figure 2:**
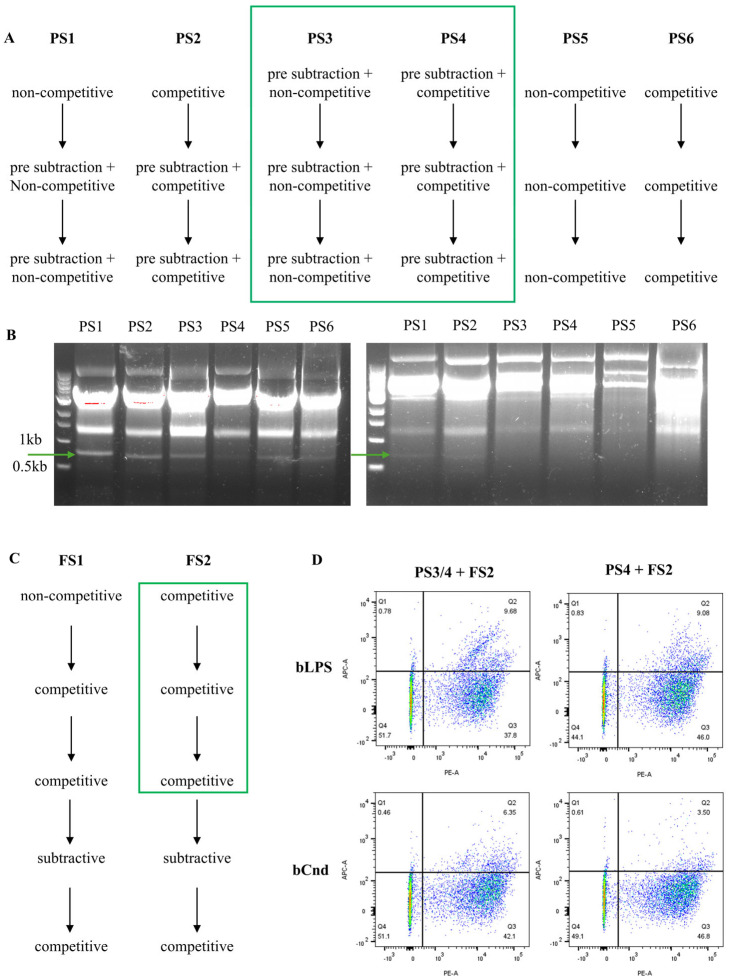
Phage and yeast selection strategies for lipid nanodisc antigens. (**A**) Schematic overview of six phage display selection strategies (PS1–PS6) used to enrich scFv binders to LPS-containing nanodiscs. Strategies incorporate non-competitive, competitive, and pre-subtractive formats designed to reduce enrichment of binders to nanodisc scaffold or non-target components (see Figure S1). The green box indicates samples that were chosen for further sampling. (**B**) Analysis of scFv insert integrity following phage selection. Plasmid DNA from second (left) and third (right) rounds of panning was digested and analyzed by gel electrophoresis to assess retention of full-length *scFv* genes (~900 bp; indicated by arrow). (**C**) Yeast display sorting strategies used to enrich antigen-binding populations. Sorting approaches included non-competitive and competitive formats to select for clones recognizing biotinylated LPS nanodiscs (bLPS). (**D**) Representative flow cytometry plots after three rounds of yeast sorting. scFv surface expression was measured using anti-SV5–PE (*x*-axis), and antigen binding was detected using streptavidin–Alexa Fluor 633 (*y*-axis). Populations corresponding to antigen-binding clones are shown.

All antibody selections utilized 180 µL of phage-displayed scFv library [30,35]. (10^12^ pfu/mL) in MSP buffer (10 mM Trizma pH 7.4 + 50 mM NaCl + 0.25 mM EDTA pH 7.4) and were pre-treated by blocking with 0.5–1% bovine serum albumin (BSA) in 1× MSP buffer for 0.5–1 h with rotation. A biotin-based labeling system was chosen for selecting antibodies against nanodiscs containing *E. coli* LPS. This allowed usage of streptavidin magnetic beads (Dynabeads M-280, ThermoFisher, #11205D, Waltham, MA, USA) and KingFisher, a magnetic bead separator system (ThermoFisher, #5400000), in our selection method. The labeling also allows subtraction with unlabeled control nanodiscs (Cnd) and specific elution of bound antibodies with unlabeled LPS nanodiscs. Various biotin-labeled and unlabeled targets were synthesized in-house (Figure 1A) and used in our antibody selection pipeline as previously described [8,14,29,30,32]. Six different phage selections with different strategies for pre-selection and competition were explored (Figure 2A). These strategies included non-competitive and competitive selections (Figure S1A). In non-competitive selections only the biotin-labeled nanodiscs were used. In competitive selections, unlabeled Cnds at 10× concentration were used to eliminate binders to nanodisc scaffold. In addition, we introduced a novel phage selection method, pre-subtraction with biotinylated Cnds (bCnd), to remove binders to the lipid layer in the nanodiscs prior to introduction of LPS nanodiscs (Figure S1A). First round phage selection used 20 µL of biotinylated LPS nanodiscs (bLPSnd) at 1 mg/mL in MSP buffer. Competitive selections included 20 µL of Cnd at 10 mg/mL (10×). Pre-subtraction selection used bCnd at 1 mg/mL in MSP buffer. The same antigen concentrations were used in first and second-round phage selections, and a 10-fold lower antigen concentration was used in the third round. MSP buffer washes were performed for each selection cycle, and no tween solutions were used, representing a departure from standard protocol. The six wash steps were 30 s for the first round and 5 min for the second and third rounds. Non-specific elution was conducted by dispersing the washed beads in 150 µL 0.1 N HCl for four minutes and neutralizing the pH with 50 µL of 1.5 M Tris pH 8.8. Specific elution was performed by incubation with excess (10×) non-biotinylated lipid nanodiscs for 30 min. Phage amplification for subsequent rounds of selection was performed as previously described [30]. Plasmid DNA from second and third-round selections were prepared and digested with restriction enzymes BssHII and NheI. Gel extraction of full-length *scFv* genes (900 bp) was performed to avoid deleted clones that may have been enriched due to hydrophobic interaction with the lipid layer of nanodiscs (Figure 2B， Figure S4). Three rounds of phage selections were performed for strategies S1–S6, and *scFv* genes were subcloned into a yeast display vector for further analysis.

Biotinylated *Y. pestis* nanodiscs (bYpnd) at 0.34 mg/mL in MSP buffer were used as the specific antigen for both the first and second round of selection. Elution with 5× unlabeled Ypnd and 0.1 M HCl was performed. Two rounds of phage panning were performed as described above.

### 2.3. ScFv Antibody Sorting by Yeast Display

Cloning into a yeast display vector was performed using restriction enzyme-based standard cloning to the pDNL6 vector system, and sorting of the yeast was performed as previously described [30]. Briefly, yeast transformation (EBY100 strain of *S. cerevisiae*) was performed using the Yeast1kit (Sigma) per the manufacturer’s instructions to obtain approximately 10^5^ diversity libraries. Transformed yeast was grown in selective media (SD/CAA), and scFv display induction was achieved using induction media (SGR/CAA). Then, 10^7^ yeast cells were incubated with labeled nanodiscs for 1 h at room temperature with rotation. Unbound antigen was washed away with MSP buffer containing 0.5% BSA (YWB MSP). The yeast display vector system includes an SV5 tag on the C terminus. Thus, scFv display levels were assessed using an anti-SV5 antibody (in-house, 1 µg/mL) conjugated to phycoerythrin (PE). The biotin label was visualized using streptavidin Alexa 633 (#S21375, ThermoFisher 5 µg/mL). The samples were washed twice, analyzed, and sorted on a BD Aria cytometer (Becton Dickinson). Three types of sorting were performed for selecting LPS antibodies (a) non-competitive sorting (biotinylated or fluorescently labeled nanodiscs with lipids), (b) competitive sorting (antigen in the presence of 2–10× unlabeled control nanodisc), and (c) subtractive sorting (labeling an undesired binder, such as a streptavidin binder, and sorting those that are unbound) (Figure 2C and Figure S1B). Subtractions with Cnd were not needed for *Y. pestis* lipid nanodisc selections. When using fluorescently labeled lipid nanodiscs, no additional labeling for antigen detection was used. Antigen concentrations varied in different rounds of sorting and ranged from 20 µL of 1 mg/mL lipid nanodiscs to 0.05 µL of fluorescently labeled nanodiscs at 0.82 mg/mL.

### 2.4. Yeast Plasmid Preparation and scFv Gene Sequencing

Plasmids from enriched yeast display libraries obtained from third- and fourth-round sorts were isolated as previously described [30] and transformed into *E. coli*. Single colonies were submitted for Sanger sequencing with PNL6 forward and reverse primers (CACTGTACTTTTAGCTCGTAC, TAGATACCCATACGACGTTC). About 400 single colonies were sequenced from various sort libraries, and the scFv sequences were analyzed to identify unique clones using Geneious^®^ Prime software (Ver 2023.2.1). Plasmids encoding unique scFvs were isolated from the bacterial colonies using Qiagen miniprep kits (#27106) per the manufacturer’s instructions and transformed back to EBY100 yeast cells for monoclonal binding assays.

### 2.5. Monoclonal Binding Assay

Monoclonal samples were prepared similarly to the yeast sorting samples described above and analyzed. Specificity in comparison to bCnd was observed. A competitive assay with 10× unlabeled lipid nanodisc (drop assay, [32]) was used to assess specificity of binding in the absence of the biotin label.

### 2.6. Affinity Maturation by Error-Prone PCR

Error-prone PCR was performed as previously described [14,32]. A yeast library with 10^7^ diversity was obtained. The yeast library sorting was performed as described above, initially using bLPSnd recognized with streptavidin-A633 for visualization of binding. The third round of sorting was performed with AF633-labeled LPS nanodiscs (633LPSnd). Upon obtaining yeast populations that showed substantial binding in comparison to secondary-only controls, the sorted yeast cells were plated to obtain single colonies. Monoclonal binding assays were performed as described above. Affinity maturation of scFvE3 that specifically recognized Ypnd was also performed using the same approach.

### 2.7. Soluble scFv-Fc Production and Binding Assays

Plasmid DNA from pDNL6 YP EP1 and EP6 was digested with restriction enzymes BssHII and NheI and subcloned into the pDNL9 human Fc vector system that secretes soluble antibody in scFv-Fc format as previously described [30,32]. After transformation, the pDNL9 clones were sequence verified and transformed into the YVH10 strain of *Saccharomyces cerevisiae*. Protein production was induced using SG/T media. Then, 1 mL of culture supernatant containing scFv-Fc was added to 10 µL of magnetic beads coated with anti-human antibody (Raybiotec #801-101-1) that were blocked with 2% BSA in MSP buffer. The scFv-Fc incubation was conducted for 1 h at room temperature with rotation. Unbound molecules were washed away using MSP buffer, and 5 µL each of 633YPnd and 633LPSnd at 0.3 mg/mL were added and incubated for 1.5 h. After washing away unbound moieties, fluorescence of magnetic beads was measured at 630 nm excitation and 660 nm emission using a Tecan Mplus spectrophotometer. The binding assay was performed in triplicate, and statistical significance was determined by a two-tailed *t*-test.

## 3. Results

### 3.1. Nanodisc Assembly

We generated nanodiscs with two sets of lipid contents (*E. coli* LPS and lipid components of *Y. pestis* membrane extract) and three types of labeling strategies (unlabeled, biotinylated, and conjugated with Alexafluor 633) as shown in Figure 1A. Nanodisc formulations were generated across a concentration range of 0.28 mg/mL to 1.24 mg/mL. Negative stain transmission electron microscopy showed the presence of disc-like particles for *Y. pestis*-derived nanodiscs (Ypnd) (Figure 1B and Figure S2A). Native PAGE analysis indicated similar migration patterns across nanodisc formulations, with no observable differences upon fluorophore labeling (Figure 1C). Dynamic light scattering measurements of Ypnd showed particle populations within the expected nanodisc size range (Figure 1D), consistent with size distributions observed across preparations (Figure S2B–D).

### 3.2. Antibody Selection Protocol for Lipid Nanodisc and Anti-LPS Binders

We chose to use a biotin-based labeling system for selecting antibodies against nanodiscs containing *E. coli* LPS. This allowed us to use streptavidin magnetic beads and the KingFisher, a magnetic bead separator system. The labeling also allowed subtraction with unlabeled empty discs (called Cnds) and specific elution of bound antibodies with unlabeled LPSnd (Figure S1A). Various biotin-labeled and unlabeled targets were synthesized in-house and used in our antibody selection pipeline. To reduce enrichment of non-specific binders, we implemented a pre-subtraction step using bCnd to remove binders to nanodisc components (Figure S1A). This strategy reduced non-specific interactions. Six different phage selections with varying combinations of pre-selection and competition were performed (Figure 2A). Initial evaluation of each strategy was performed by restriction digestion to assess the presence of the expected 900 bp scFv fragment. Agarose gel images (Figure 2B) showed that second-round phage outputs for all six selection strategies contained the expected fragment; however, only PS1 and PS2 strategies retained detectable full-length scFv bands after the third round of selection. The gel images also showed the presence of smaller plasmids, necessitating gel extraction of the approximately 900 bp fragment. The first-round phage titers ranged from 10^4^–10^5^, the second round 10^6^–10^8^, and third-round titers approached 10^8^ without clear differentiation between strategies. Eluted phage titers from the second round, which showed the most differentiation between selection samples, are given in Supplementary Table S1.

We proceeded with the samples showing strong full-length scFv representation. Thus, the selection outputs for second-round PS1–PS6 and third-round PS1 and PS2 were subcloned into the yeast display vector pDNL6, and flow cytometry-based sorting with and without Cnd subtraction was performed (Figure 2C). We analyzed sort populations for specificity to bLPSnd and compared them to bCnd and secondary-only controls. The highest percentage of specific populations in Q2 was observed in yeast populations derived from phage selection strategies PS3 and PS4. Enrichment of antigen-binding populations was highest in sort 3, which included excess unlabeled Cnd during staining (Figure 2D).

**Figure 3:**
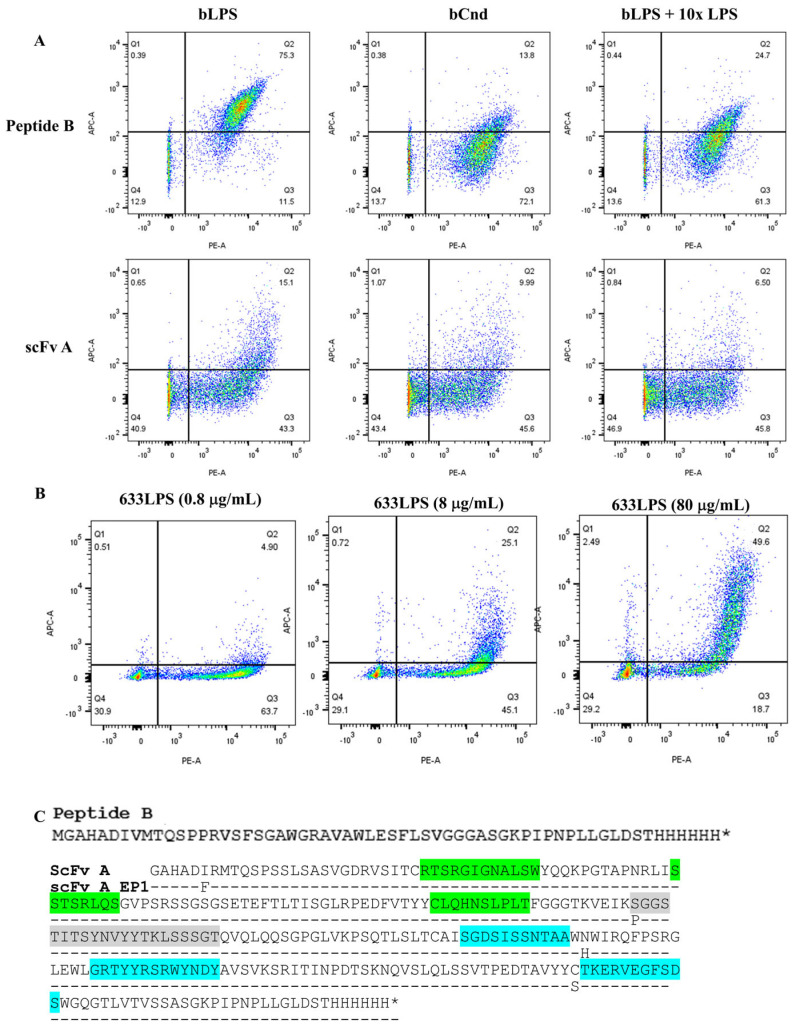
A peptide and scFv antibody recognizing an LPS nanodisc. (**A**) One 60-amino-acid peptide (Peptide B) and one full-length scFv (scFv A) identified from in vitro antibody selection that bind biotinylated LPS nanodiscs (bLPSnd). Representative flow cytometry plots show binding to bLPSnd, comparison to biotinylated control nanodiscs (bCnd), and competition with excess unlabeled LPS nanodiscs. The scFv surface expression was measured using anti-SV5–PE (*x*-axis), and antigen binding was detected using streptavidin–Alexa Fluor 633 (*y*-axis). (**B**) Binding profile of affinity-matured scFv A-EP1 using Alexa Fluor 633 (A633)-labeled LPS nanodiscs (633LPSnd). Antigen binding was detected by direct fluorescence of labeled nanodiscs (*y*-axis), and scFv expression was measured using anti-SV5–PE (*x*-axis). (**C**) Amino acid sequences of the two selected binders. Variable light chain CDRs are highlighted in green, variable heavy chain CDRs in teal, and the linker region in grey.

Plasmid DNA from these sort outputs was prepared and transformed into *E. coli* for DNA sequencing. Analysis of ~100 clones identified four different sequences: two unique scFvs and two peptide sequences. Monoclonal analyses confirmed specific recognition of LPSnd by one peptide (Peptide B) and one scFv (scFv A). Data in Figure 3A show that peptide B recognizes bLPSnd and does not bind bCnd. Binding was reduced in the presence of 10× unlabeled LPS nanodiscs, consistent with antigen-specific recognition, rather than interaction with the biotin label. The recognition signal for scFv A shows a similar pattern (Figure 3A); however, specific binding is comparatively low. To establish all possible protocols in this LPS system and to improve this binder, we proceeded with affinity maturation. During affinity maturation, Alexa Fluor 633 (A633)-labeled LPS nanodiscs (633LPSnd) were introduced as the antigen. The 633LPSnd nanodiscs showed strong, concentration-dependent binding (Figure 3B). Affinity maturation resulted in an increase in binding signal 1.6-fold from the original scFv A (Mean Fluorescent Intensity [MFI] = 2051) to scFv A EP1 (MFI = 3251). Sequence comparison showed that four amino acid substitutions were associated with this difference (Figure 3C). These results supported application of the optimized workflow to *Y. pestis* nanodisc selections.

### 3.3. Antibodies to Yersinia Pestis Lipid Nanodiscs

**Figure 4:**
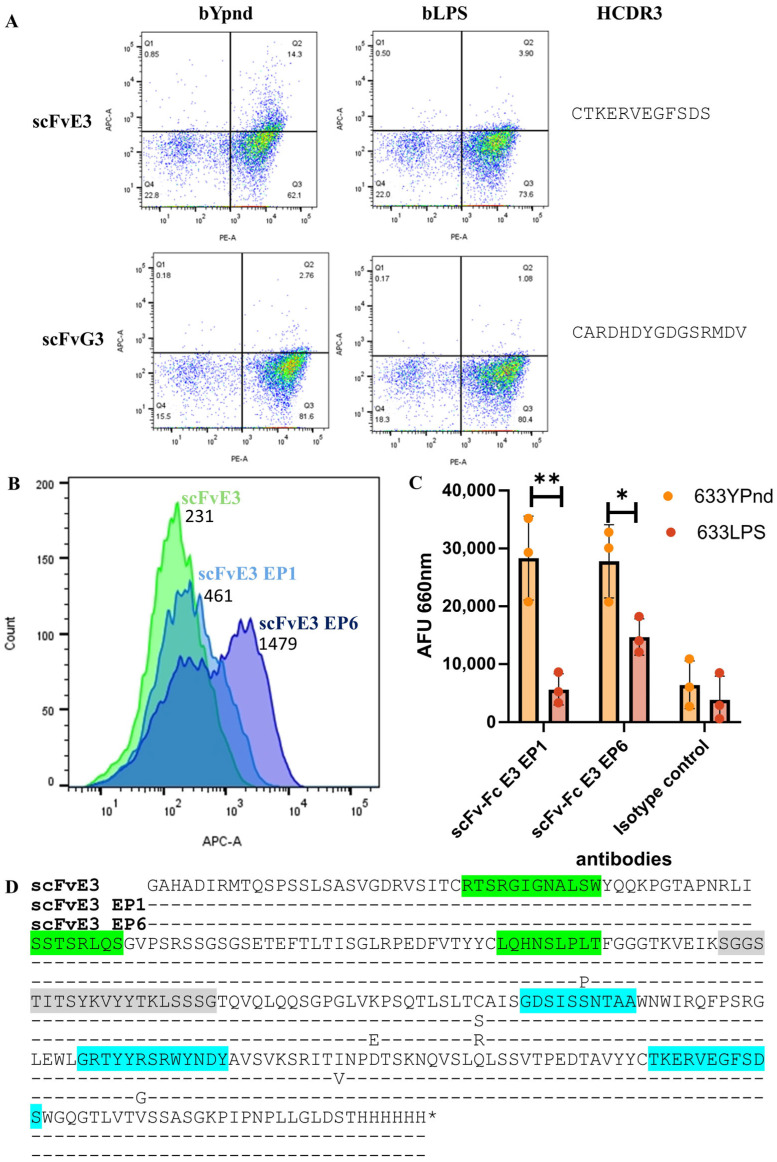
The scFv antibodies that recognize nanodiscs containing *Y. pestis* membrane lipids. (**A**) Representative flow cytometry plots showing binding of scFvE3 and scFvG3 to biotinylated *Y. pestis* nanodiscs (bYpnd) compared to biotinylated LPS nanodiscs (bLPSnd). The scFv surface expression was measured using anti-SV5–PE (*x*-axis), and antigen binding was detected using streptavidin–Alexa Fluor 633 (*y*-axis). (**B**) Binding profiles of affinity-matured variants of scFvE3 (EP1 and EP6) using Alexa Fluor 633-labeled *Y. pestis* nanodiscs (633Ypnd). Antigen binding was detected by direct fluorescence (*y*-axis), and scFv expression was measured using anti-SV5–PE (*x*-axis). Mean fluorescence intensity (MFI) values are indicated. (**C**) Binding analysis of soluble scFv-Fc antibodies. Secreted scFv-Fc proteins were captured on anti-human Fc magnetic beads and incubated with Alexa Fluor 633-labeled nanodiscs (633Ypnd and 633LPSnd). Binding was measured by fluorescence intensity. (**D**) Amino acid sequences of parental and affinity-matured variants of scFvE3. Variable light chain CDRs are highlighted in green, variable heavy chain CDRs in teal, and the linker region in grey.

For antibody selections against *Y. pestis,* we applied the framework developed in LPS selections to optimize selection efficiency while minimizing nanodisc usage. First, we performed three rounds of phage selections, implementing quality control to monitor retention of full-length scFvs with each round. Full-length *scFv* genes from second- and third-round selections were subcloned into yeast display and subjected to flow cytometry-based analysis and sorting. Sequencing of 96 clones from binding populations showed two dominant scFv sequences. These clones (scFvE3 and scFvG3) were assayed for specificity of binding to bYpnd in comparison to bLPSnd. The binding pattern and heavy chain complementarity-determining region 3 (HCDR3) sequence for both clones are shown in Figure 4A. As scFvE3 showed higher binding signal, it was chosen for affinity maturation. Data in Figure 4B show an approximately 2-fold increase in binding signal for scFvE3 EP1 and a 6.4-fold increase for scFvE3 EP6. These two clones were further subcloned into a yeast secretion vector to express soluble antibodies in scFv-Fc format. Secreted scFv-Fc proteins containing a human Fc domain were captured on magnetic beads coated with anti-human antibodies and incubated with Alexa Fluor 633-labeled *Y. pestis* nanodiscs (633Ypnd) and 633LPSnd. The binding data for soluble antibodies in Figure 4C show preferential binding to *Y. pestis* nanodiscs for both scFvs, with scFvE3 EP1 showing higher binding signal. Sequence analysis (Figure 4D) showed that differences in binding signal were associated with two amino acid substitutions in scFvE3 EP1 and five substitutions in scFvE3 EP6. Sequence comparison showed similarity between these scFvs and scFv A EP1 identified from LPS selections, though differences were seen in LCDR3 and HCDR3 regions (Figure S3A), and the second scFv isolated from Ypnd selections (scFvG3) differed substantially from scFvE3 (Figure S3B).

## 4. Discussion

Antibodies directed against membrane lipids are recognized for their roles in antimicrobial immunity and autoimmune pathogenesis [36]. Lipid-reactive antibodies are predominantly of the IgM isotype [37], functioning as an early defense mechanism. Phage display–based approaches have been used to isolate lipid-binding antibodies, including selections against synthetic 15-ketocholestane [36] and purified methyl palmitate [38] using immobilized antigen formats. Molecular dynamics simulations have provided mechanistic insight into lipid-binding antibodies with HIV-neutralizing activity [39], and recent advances in computational protein design [40] have the potential to expand the repertoire of engineered lipid binders. Nanodiscs have also emerged as effective platforms for presenting membrane-associated antigens, as demonstrated in antibody selection against bacteriorhodopsin [41]. In this study, we developed a nanodisc-based platform for the selection of bacterial lipid-targeting scFv (IgG-class) antibodies.

In our previous work, we described a standardized method for preparing nanodiscs containing bacterial lipids from the *Y. pestis* cell membrane [33]. Here, we explored labeling strategies to enable in vitro antibody selections. Using unlabeled, biotinylated, and Alexa 633–labeled nanodiscs enabled evaluation of multiple phage panning and yeast sorting strategies. Nanodisc formulations (control, LPS, *Y. pestis*) showed consistent size characteristics across labeling conditions (Figure 2 and Figure S2). Evaluation of labeling effects using *E. coli* LPS nanodiscs informed selection conditions that were subsequently applied to *Y. pestis* lipid nanodiscs.

Performing antibody selections on *Y. pestis* lipids was expected to be challenging for the following reasons: (1) purified lipids from this bacterium are not commercially available, (2) identification of lipids incorporated into nanodiscs and reproducibility of nanodisc assembly are difficult to ascertain, (3) lipids are not amenable to the same chemical modifications that allow for proteins to be tethered to a surface during selections [42], and (4) lipid nanodiscs may be sensitive to temperature and handling.

To address these challenges, we used nanodiscs containing commercial *E. coli* LPS to develop the antibody selection methodology. Pre-subtraction with control nanodiscs reduced enrichment of non-specific binders during phage selection (Figure 2A). Pre-incubation with bCnd followed by removal of bound phage improved selection specificity (Figure S1A). Two rounds of selection were used for these lipid-based antigens, as performing a third round reduced the proportion of full-length scFvs (Figure 2B). This reduction is consistent with preferential amplification of truncated clones during overnight bacterial culture. Detergents were excluded from wash buffers due to potential sensitivity of lipid-based antigens, and gentle handling conditions were used.

During the phage selection process, the elution step produced unexpected results (Supplementary Table S1). Acid elution (HCl) generally yields higher phage titers than specific elution with excess unlabeled LPS nanodiscs (10×) because acid treatment releases *all* bead-associated phage, including specific, non-specific, and truncated clones. In contrast, specific elution is expected to recover only antigen-specific binders. However, higher titers were observed following specific elution. This may reflect enrichment of clones with hydrophobic interactions with lipid nanodiscs.

During flow cytometry–based analysis of yeast clones, competitive sorting, where yeast-displayed antibodies were first exposed to 2–10× unlabeled control nanodiscs for 30–60 min prior to addition of bLPSnd, was effective for reducing enrichment of non-specific binders (Figure 2C and Figure S1B). We also attempted to remove non-specific binders by sorting yeast that did not bind bCnd (sort Q4); however, this strategy was not successful, as we did not identify specific binders in the subsequent sorts. Similar to phage selection, three rounds of yeast sorting were sufficient to obtain enriched populations (Figure 2D). This observation is consistent with preferential amplification of truncated clones during overnight culture. During yeast sorting experiments, the nanodiscs were structurally fragile and required careful handling. Vigorous mixing methods, such as vortexing or rapid pipetting, compromised nanodisc integrity and were therefore avoided. Samples were therefore handled using gentle mixing to preserve nanodisc integrity. Additionally, nitrocellulose-containing filter plates commonly used for monoclonal analysis were unsuitable for lipid nanodisc–based assays, as the nanodiscs appeared to destabilize upon contact with nitrocellulose.

Despite efforts to reduce deleted clones, one of the strongest binders to LPS obtained in our selection campaign was a 60 amino acid peptide (peptide B, Figure 3A,C). In our yeast display experiments, this peptide consistently recognized LPS nanodiscs across multiple experiments. Binding was reduced in the presence of control nanodiscs (bCnd) and competing unlabeled LPS nanodiscs, consistent with selective recognition. The second binder was a full-length scFv (scFv A, Figure 3A,C). Binding behavior for scFv A showed a similar pattern, with reduced signal in the presence of control nanodiscs and competing antigen. The low binding signal for scFv A provided an opportunity for optimization through error-prone PCR-based affinity maturation and subsequent sorting of higher-affinity variants.

Our protocols for affinity maturation were based on previous work [29,32] that established the power of error-prone PCR to introduce mutations to *scFv* genes and the ability of flow cytometry-based sorting to isolate high-affinity binders from diverse libraries. With this protocol, we observed increased binding signal for both scFv A that recognize *E. coli* LPS nanodisc and scFvE3 (*Y. pestis* lipid nanodiscs). As shown in Figure 3B and Figure 4B, this process resulted in changes in binding signal of approximately 1.6- and 6.4-fold. This may be a reflection of increased scFv expression labeling, avidity, or assay format. To minimize the possible effect of variation in labeling of the antigen and antibody expression and assay conditions, experiments comparing original and affinity-matured scFv constructs were performed in parallel. Evaluation of affinity maturation coincided with the availability of fluorescent nanodiscs. These nanodiscs produced a strong signal, such that lower limits of detection were difficult to define. Even low antigen concentrations (0.5 µL antigen into a total volume of 10 mL) produced strong signal, whereas the biotinylated nanodiscs showed weaker signal at higher concentrations. These properties may facilitate improved visualization and sorting of binding populations. Fluorescent nanodiscs also reduce the likelihood of selecting streptavidin-binding clones, improving the signal-to-noise ratio and enhancing sorting efficiency. To our knowledge, this represents an early application of Alexa 633-labeled nanodiscs, which provided advantages for detection and sorting.

Non-specific binders were of concern in this work, due to the possibility of hydrophobic interactions. High background levels seen in phage display demonstrated this concern, but the strengths of yeast display and sorting by flow cytometry allowed us to eliminate binders to components of the nanodiscs, including MSP, nanodisc surface, biotin, or streptavidin. In order to validate specificity of binding, our monoclonal binding assays used bCnd (Figure 3A) and bLPS (Figure 4A) as controls to demonstrate specificity to target nanodiscs.

While the signal strength differed between biotinylated and fluorescent nanodiscs, the ability to switch between formats without disrupting binding suggests that nanodisc preparation did not substantially alter antibody recognition. Across selections and sorting experiments, no major batch-to-batch variability was observed, consistent with reproducible nanodisc preparation.

Sequence comparison of the LPS-binding scFv A and its affinity-matured variant scFv A EP1 revealed that the canonical CDR sequences, including VH CDR2 and VH CDR3, were unchanged, indicating that the enhanced binding signal observed for EP1 is unlikely to arise from direct remodeling of the dominant antigen-contact loops [43]. Instead, EP1 contains substitutions within the interdomain linker and heavy-chain framework that may influence paratope presentation indirectly. Notably, a serine-to-proline substitution within the Gly/Ser-rich VL–VH linker may alter linker flexibility and interdomain orientation. Such changes could influence the relative positioning of VL and VH domains and thereby affect apparent binding. In addition, EP1 carries a cysteine-to-serine substitution within the conserved VH VYYC motif proximal to the CDR3 region. This substitution may affect VH structural stability or local conformational dynamics, which could influence antigen binding. Finally, an asparagine-to-histidine substitution near the VH CDR1 boundary introduces a titratable side chain that may alter hydrogen-bonding geometry and potential pH-sensitive electrostatic interactions, which may contribute to binding differences [44]. Collectively, these findings are consistent with the possibility that the increased binding signal of scFv A EP1 arises not from primary sequence changes within HCDR3 but from framework and linker-associated effects that influence CDR positioning. These observations suggest that affinity maturation can modulate binding through subtle structural tuning of domain orientation and loop dynamics, even in the absence of direct alterations to the core CDR sequences.

Sequence comparison of the affinity-matured *Y. pestis* binding variants EP1 and EP6 with the parental scFv E3 showed that the majority of framework and CDR residues are conserved, with mutations clustered in discrete regions. Notably, HCDR3, frequently a dominant determinant of antigen specificity [43], remained unchanged across all three constructs, which could indicate that mutations in HCDR3 reduced binding. The scFvE3 EP1 retains identical CDR sequences to the parental clone but introduces framework mutations in the heavy chain, including substitution of the conserved VH cysteine (C→S). This residue typically participates in the intradomain disulfide bond that stabilizes the variable heavy chain. Substitution at this position may influence domain stability or CDR positioning, potentially affecting antigen binding. In EP6, a substitution within light-chain CDR3 (VL CDR3), L→P (LQHNSLPLT to LQHNSLPPT), represents the only direct change within a light-chain antigen-contact loop. By forming a proline–proline pair, this substitution may alter local loop geometry and influence CDR3 conformation. EP6 also contains a VH CDR2 substitution (R→G within the GRTYYRSRWY motif), possibly altering electrostatic complementarity and increasing local backbone flexibility within the paratope. An additional C→R substitution in EP6 may contribute to differences in expression or stability, consistent with broader binding distribution observed for scFvE3 EP6 (Figure 4D). Taken together, these data are consistent with the possibility that affinity maturation in this series did not rely on changes within HCDR3 but instead involved modulation of light-chain CDR3 conformation (EP6), alteration of VH CDR2 chemistry (EP6), and framework-mediated effects on VH structural stability (EP1). These observations suggest that affinity optimization can occur through changes outside of HCDR3, including modifications in other CDRs and framework regions. Further analysis of additional variants, including scFvG3 (Figure S3B), may provide additional insight into sequence–function relationships.

The amino acid sequences of single-chain antibodies binding nanodiscs containing *E. coli* LPS and *Y. pestis* lipids showed notable similarity. We compared the sequences of scFv A EP1 and scFvE3 EP1 (Figure S3A). Although sequence similarity could suggest broader lipid reactivity, our specificity data show that the binders are distinct. ELISA analysis (Figure 4C) showed differential binding between the affinity-matured variants, with scFvE3 EP1 exhibiting strong binding to 633Ypnd and reduced reactivity toward 633LPSnd, while scFvE3 EP6 showed comparatively lower signal overall and reduced selectivity. Both antibodies produced signals significantly above the isotype control, consistent with antigen-dependent binding, with higher signal observed for 633Ypnd, particularly for EP1. These results are consistent with increased selectivity of EP1 toward *Y. pestis* nanodiscs relative to *E. coli* LPS nanodiscs. The sequence similarity raises the possibility that shared lipid components, such as LPS, may contribute to binding of scFvE3. The LPS found within *Y. pestis* differs from *E. coli* in meaningful ways, including the fact that *Y. pestis* has a rough-type LPS that does not contain O-antigen [45]. These structural differences may contribute to the observed selectivity.

Sequence comparison (Figure S3A) identified seven amino acid differences between these two antibodies. These observations suggest that the specificity shift does not arise from alterations in canonical CDR3 motifs, which remain conserved, but rather from framework and interdomain modifications that may influence paratope geometry. In particular, substitutions affecting the VL–VH linker and conserved VH cysteine positions may affect interdomain orientation and CDR positioning, which could influence binding to membrane-presented epitopes. The preferential binding of *Y. pestis* nanodiscs by scFv E3 EP1 is consistent with changes in paratope presentation rather than wholesale remodeling of antigen contact residues. Collectively, these observations suggest that affinity maturation can modulate membrane target specificity through changes outside canonical CDR regions.

Though our goal was to pursue antibodies against *Y. pestis*, optimization using LPS nanodiscs informed subsequent selections. Whereas LPS-based antibody selection took approximately 2.5 years, selections against *Y. pestis* nanodiscs progressed within 9 months, reducing experimental time and use of *Y. pestis* reagents. One improvement to our process was implementation of a quality-control step to enrich for full-length scFv sequences during phage selection. This reduced background signal and downstream processing requirements. Fluorescent nanodiscs also contributed to improved detection efficiency. This workflow may be adaptable to nanodiscs derived from other pathogens. Differences in selection efficiency may reflect the properties of the lipid mixtures used; however, further characterization would be required to identify specific antigenic components.

Future work could evaluate the functional effects of lipid-targeting antibodies during *Y. pestis* infection. These experiments could include IgG-based validation of binding to lipid nanodiscs, complemented with orthogonal approaches (e.g., lipid binding arrays, surface plasmon resonance) to define specificity. To further characterize these antigens, liquid chromatography–mass spectrometry analysis has been performed, and evaluation of the results is in progress. Affinity maturation improved binding signals for selected clones, and additional rounds of optimization could further refine scFv A EP1 and scFvE3 EP1.

The results demonstrate that antibodies can be selected against bacterial lipid-containing nanodisc antigens. The methodology also highlights challenges associated with generating specificity toward complex lipid mixtures. Recently, Biryukov et al. demonstrated the vaccine efficacy of nanodiscs containing recombinant protein antigens LcrV and F1 of *Y. pestis* using BALB/c mice [46]. Together, these findings support further investigation of nanodisc-based antigen presentation systems, including those incorporating lipid components, for antibody discovery and vaccine design.

## 5. Conclusions

In this study, we developed and applied a nanodisc-based platform for in vitro selection of antibodies targeting bacterial lipid-containing antigens. Using optimized phage and yeast display strategies, we identified peptide and scFv binders to LPS and *Y. pestis*-derived nanodiscs and demonstrated that affinity maturation can enhance binding without requiring changes to the canonical CDR regions. These findings establish a framework for antibody discovery against membrane-associated lipid antigens and highlight both the feasibility and challenges of targeting complex lipid mixtures.

## Data Availability

The original contributions presented in this study are included in the article/supplementary material. Further inquiries can be directed to the corresponding author.

## Supplementary Materials

The following supporting information can be downloaded at: https://www.mdpi.com/article/10.3390/pathogens15060651/s1, Table S1: Phage selection strategy and eluted phage titers for second round; Figure S1: Phage selection and yeast sorting strategies; Figure S2: Characterization of LPS and *Y. pestis* nanodiscs; Figure S3: ScFv sequence comparison. Figure S4：Original image of Figure 2B.

## Abbreviations

The following abbreviations are used in this manuscript:

bLPS	Biotinylated LPS
bLPSnd	Biotinylated LPS nanodiscs
bYpnd	Biotinylated *Y. pestis* nanodiscs
CDR	Complementarity-determining region
Cnd	Control nanodiscs
DLS	Dynamic light scattering
F1	Fraction 1
Fc	Fragment crystallizable
FS(1,2)	Flow Sort 1, 2
HCDR3	Heavy-chain complementarity-determining region 3
HDL	High-density lipoprotein
LCDR3	Light-chain complementarity-determining region 3
LcrV	Low-calcium response v
LPS	Lipopolysaccharide
PS(1-6)	Phage selection 1–6
scFv	Single-chain variable fragment
V_H_	Variable heavy chain
V_L_	Variable light chain
Ypnd	*Y. Pestis* nanodiscs
