# Supplementary material for: In Vitro Selection of Antibodies Targeting Yersinia pestis Membrane Lipids Using Nanodisc-Based Antigen Presentation
Source: Pathogens. 2026 Jun 20;15(6):651. doi: 10.3390/pathogens15060651 (PMC13304831; doi:10.3390/pathogens15060651)
Supplement: Supplementary file 1 [file pathogens-15-00651-s001.zip › supplementary Figure 4.pdf]

Gel images Figure 2B

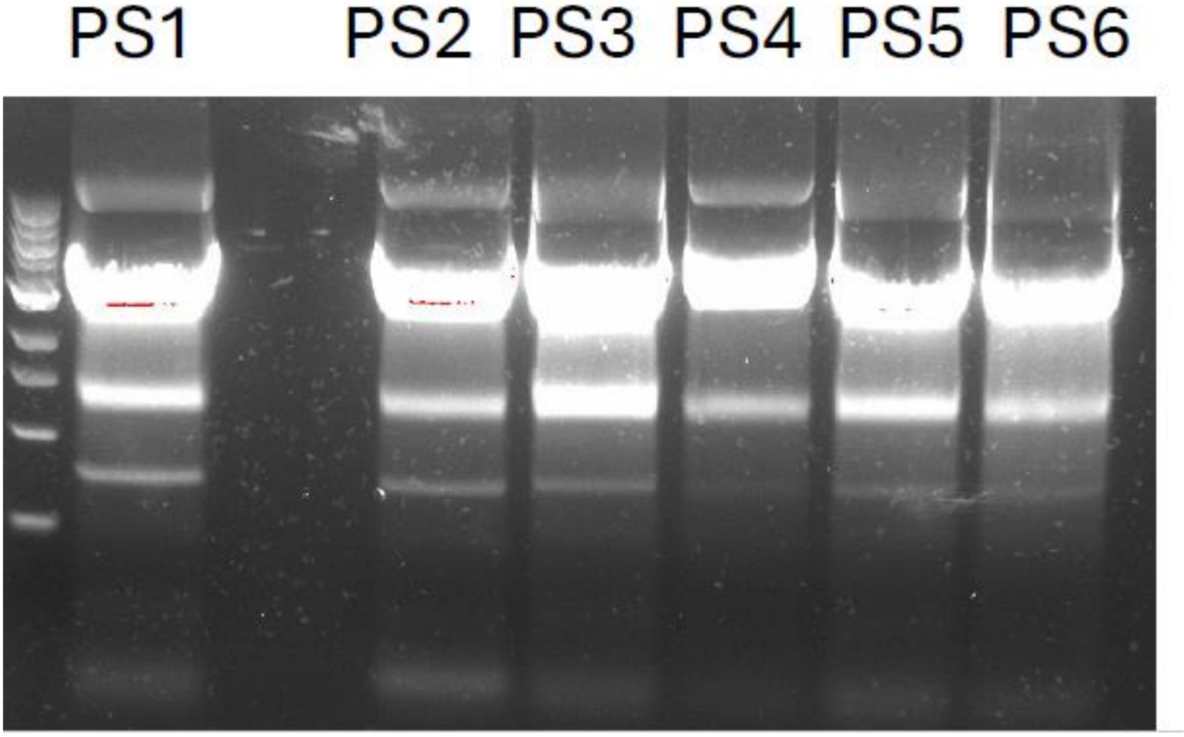

Figure S4： Original image of Figure 2B. Figure 2B: Gel extraction of full-length scFv genes (900 bp) was performed to avoid deleted clones that may have been enriched due to hydrophobic interaction with the lipid layer of nanodiscs.
