# Supplementary material for: In Vitro Selection of Antibodies Targeting Yersinia pestis Membrane Lipids Using Nanodisc-Based Antigen Presentation
Source: Pathogens. 2026 Jun 20;15(6):651. doi: 10.3390/pathogens15060651 (PMC13304831; doi:10.3390/pathogens15060651)
Supplement: Supplementary file 1 [file pathogens-15-00651-s001.zip › Supplementary Tables.pdf]

Supplementary Tables

Supplementary Table S1: Phage selection strategy and eluted phage titers for second round

Phage selection strategy	Antigen	Eluted phage titer
S1	b-LPS nanodisc	$4.0x10^{6}$
	Streptavidin beads only	$4.1x10^{6}$
S2	b-LPS nanodisc	$2.4x10^{6}$
	Streptavidin beads only	$1.2x10^{6}$
S3	b-LPS nanodisc	$32.2x10^{6}$
	Streptavidin beads only	$6.4x10^{6}$
S4	b-LPS nanodisc	$4.1x10^{6}$
	Streptavidin beads only	$2.8x10^{6}$
S5	b-LPS nanodisc	$6.8x10^{6}$
	Streptavidin beads only	$7.2x10^{6}$
S6	b-LPS nanodisc	$3.2x10^{6}$
	Streptavidin beads only	$10.1x10^{6}$
