# Supplementary figures and images for: In Vitro Selection of Antibodies Targeting Yersinia pestis Membrane Lipids Using Nanodisc-Based Antigen Presentation
Source: Pathogens. 2026 Jun 20;15(6):651. doi: 10.3390/pathogens15060651 (PMC13304831; doi:10.3390/pathogens15060651)

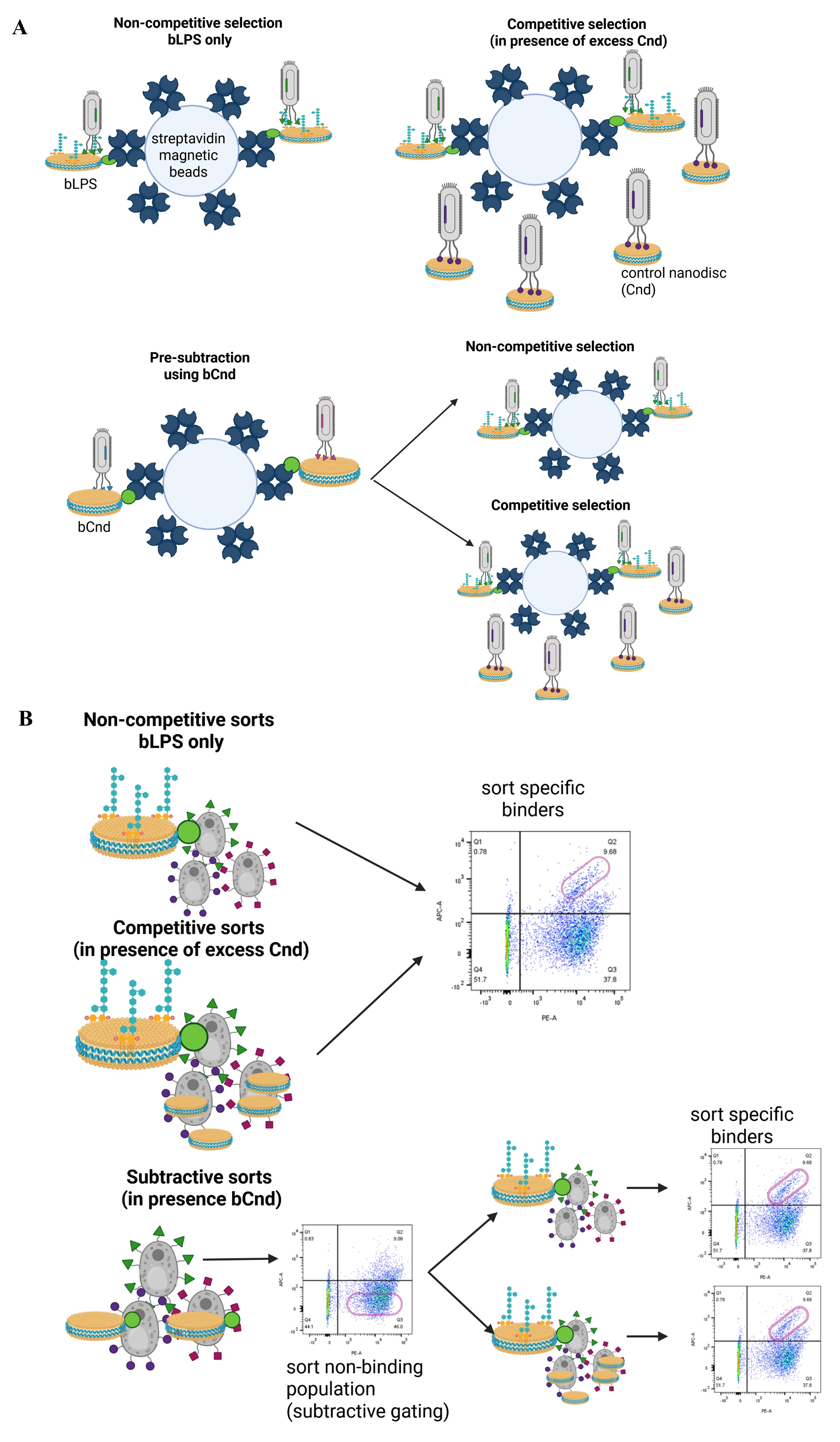

Supplement: Supplementary file 1 [file pathogens-15-00651-s001.zip › Supp Fig 1.TIF]

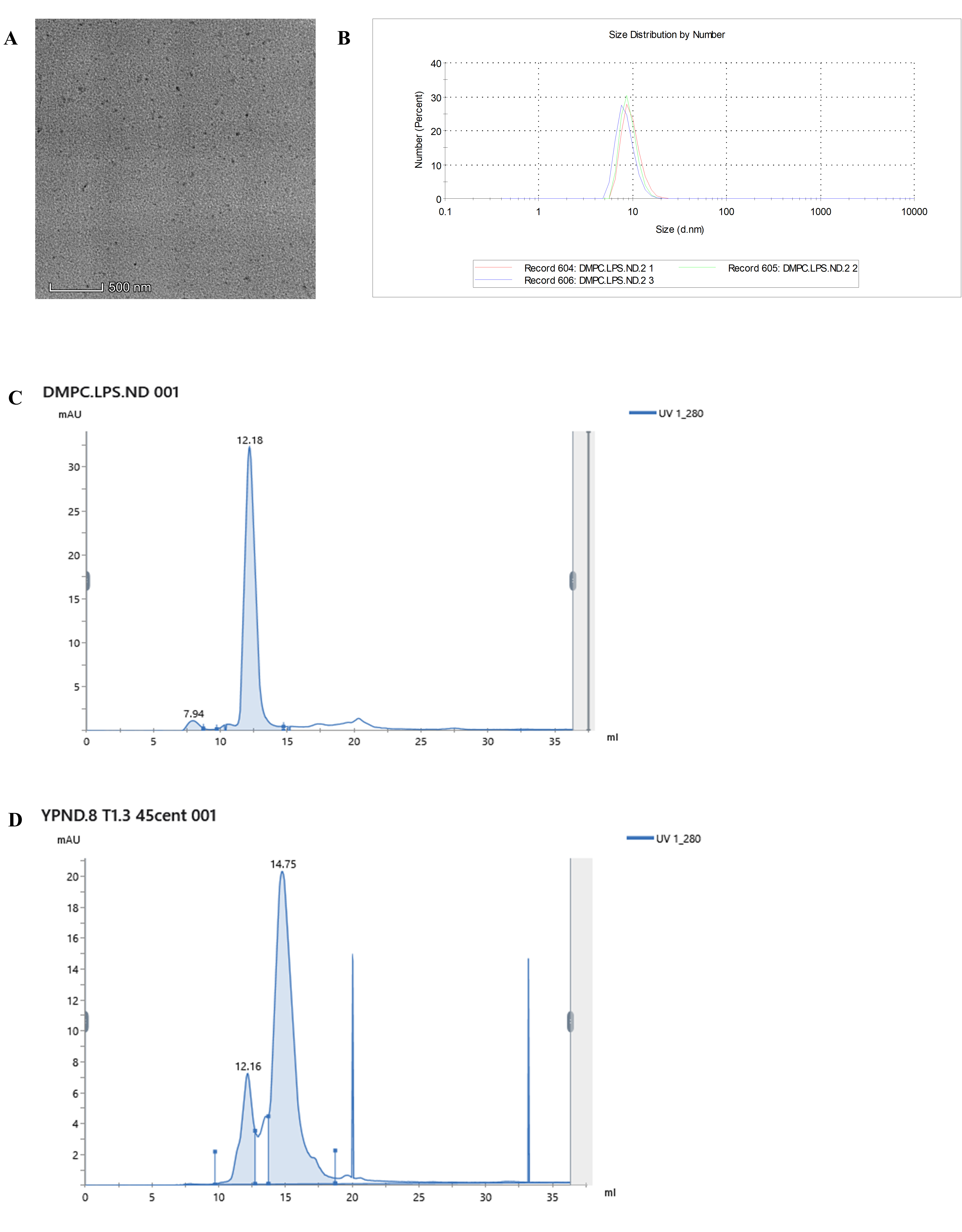

Supplement: Supplementary file 1 [file pathogens-15-00651-s001.zip › Supp Fig 2.TIF]

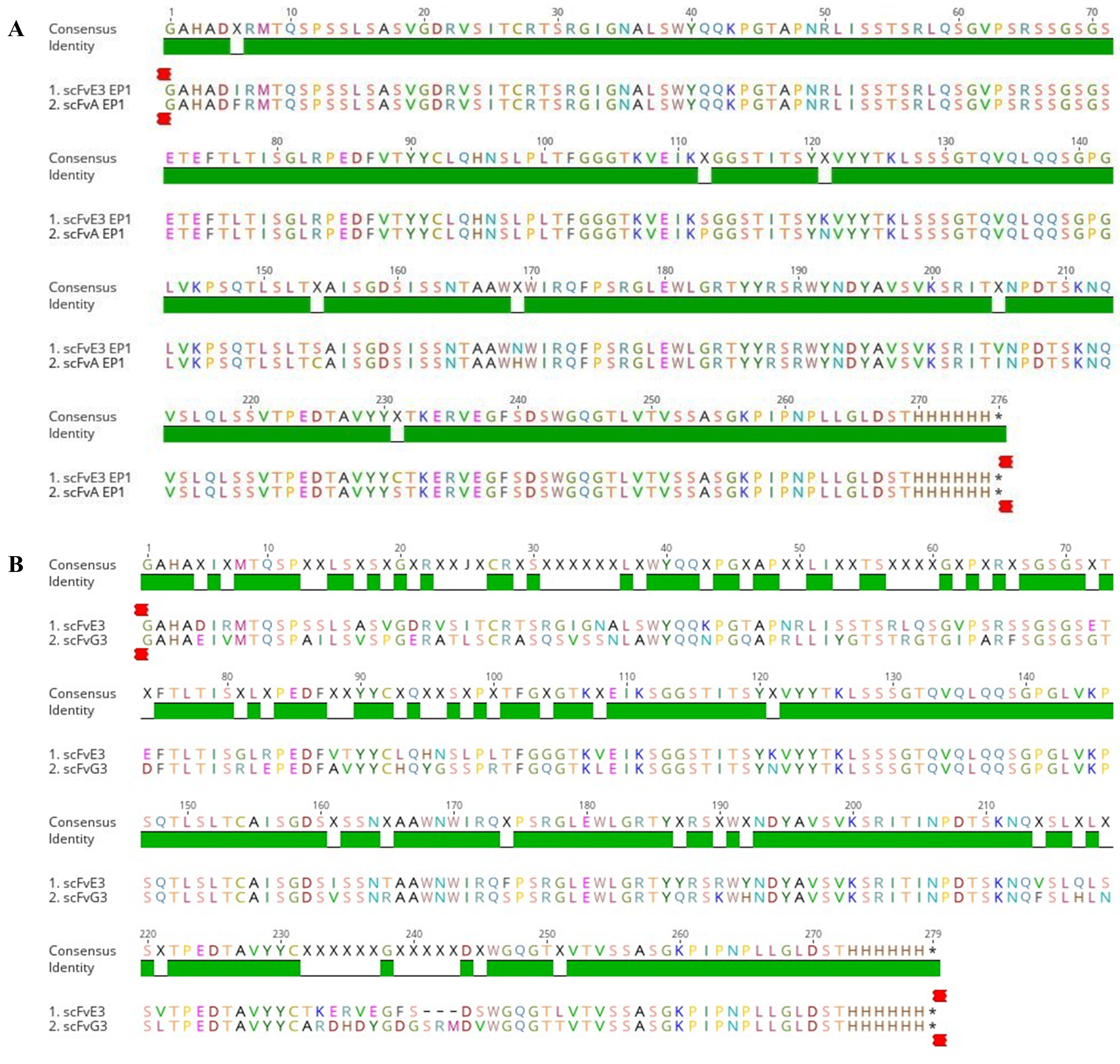

Supplement: Supplementary file 1 [file pathogens-15-00651-s001.zip › Supp Fig 3.TIF]
